# Source–sink characterization, classification and responses to nitrogen in different wheat cultivars

**DOI:** 10.3389/fpls.2026.1817057

**Published:** 2026-04-20

**Authors:** Siqi Zhang, Shaoyu Han, Yandong Yang, Jie Zhang, Guoqiang Li, Zhongwei Tian

**Affiliations:** 1 Institute of Agricultural Information Technology, Henan Academy of Agricultural Sciences, Zhengzhou, China; 2 Huanghuaihai Key Laboratory of Intelligent Agricultural Technology, Ministry of Agriculture and Rural Areas, Zhengzhou, China; 3 Key Laboratory of Crop Physiology, Ecology and Production Management, Ministry of Agriculture/College of Agriculture, Nanjing Agricultural University, Nanjing, China

**Keywords:** grain yield, nitrogen regulation, source-sink classification, source-sink relationship, wheat

## Abstract

Optimizing source-sink dynamics is fundamental to understanding crop yield formation and achieving high productivity. This study aims to elucidate the physiological mechanisms by which source-sink traits influence wheat yield and to classify cultivars based on their limiting factors. Field experiments were conducted using three wheat cultivars (YM1, YM25, and ZM27) under two nitrogen (N) application rates to assess their source-sink characteristics. Compared with YM1, the cultivars YM25 and ZM27 exhibited significantly enhanced source capacity, characterized by larger leaf area, higher specific leaf weight (SLW), extended SPAD duration, and photosynthetic duration. Consequently, YM25 and ZM27 achieved significantly higher grain yields and sink capacities. The yield advantage was primarily driven by an increased number of kernels per spikelet and higher 1000-kernel weight (TKW), which resulted from a faster grain-filling rate, earlier onset of peak filling, and improved grain fullness. In contrast, YM1 displayed limited source strength (smaller leaf area, lower SLW) and poor sink activity, characterized by slower grain filling and reduced grain weight. Source-sink classification analysis revealed that YM25 and ZM27 are source-limited cultivars, possessing high source and sink strengths but a relatively high sink-source ratio. Conversely, YM1 is a sink-limited cultivar, exhibiting low source and sink strengths with a relatively low sink-source ratio. Furthermore, N fertilization effectively regulated source-sink relationships, specifically by mitigating source limitations in high-yielding cultivars. These findings provide a theoretical basis for cultivating high-yield wheat varieties by coordinating source-sink interactions.

## 1 Introduction

Wheat (*Triticum aestivum L.*) is one of the most extensively cultivated staple crops worldwide. In China, it ranks second only to rice and maize, serving as a key component of national food security (Zhang et al., 2010). With the global population projected to exceed 9 billion by 2050, a substantial increase in wheat productivity is imperative to meet escalating food demands (Alonso et al., 2018; Asseng et al., 2017). Although strategic initiatives, such as China’s “No. 1 Central Document,” emphasize stabilizing grain-sown areas and maintaining annual output above 650 million tons, food security remains a critical global challenge amidst sustained population growth and compounding stresses, including malnutrition and climate extremes. Consequently, enhancing grain yield has become a central objective in modern crop science (Long et al., 2015).

The concepts of “source” and “sink” in photosynthetic production, first introduced by Mason and Maskell (1928), have become fundamental to analyzing crop yield formation. In wheat, the source refers to organs that produce or export assimilates, including green tissues such as stems, sheaths, and leaves, which provide the material basis for grain formation (Fabre et al., 2020). The sink comprises organs that absorb and utilize these assimilates, primarily the developing grains (Gao et al., 2021). Grain yield is essentially determined by the balance between the supply of photosynthates (source activity) and the storage capacity of grains (sink strength) (Smith et al., 2018; Yu et al., 2015). The coordination between source and sink is modulated by multiple factors, including plant architecture, climatic conditions, and fertilization strategies (Li et al., 2022; Zhang et al., 2010).

Agronomic management and breeding history have significantly shaped source-sink dynamics. For instance, ideal “super wheat” plant types are characterized by erect upper leaves to optimize light interception (Fahad et al., 2019). Furthermore, nitrogen (N) fertilization serves as a key nutrient regulating assimilate production and indirectly influencing source-sink dynamics (Duan et al., 2019). Significant differences in source-sink traits have been observed under different N regimes (Li et al., 2022). Studies have demonstrated that N deficiency during mid-to-late developmental stages can restrict leaf area expansion, reduce photosynthetic efficiency, and hinder dry matter allocation to reproductive organs (Kaduwal et al., 2024). Additionally, the “Green Revolution” significantly improved source-sink dynamics through the introduction of semi-dwarf *Rht* genes, which reduced plant height while increasing kernels per spike and grain weight (Reynolds et al., 2009; Xiao et al., 2012).

Despite extensive research, the primary limiting factors for wheat yield—whether source or sink—remain a subject of debate. Some studies, such as Serrago et al. (2013), found that yield was predominantly sink-limited under most conditions, whereas others suggest that both source and sink jointly limit yield (Boussakouran et al., 2021). Conversely, recent research indicates that modern wheat cultivars have achieved significant improvements in sink size, rendering source limitations more pronounced as the key constraint on yield (Gao et al., 2021; Zhang et al., 2016). These inconsistencies may stem from the diverse genetic backgrounds of tested cultivars, which complicates the accurate definition of functional source-sink relationships. Regardless of whether the limitation is source- or sink-based, both factors affect yield formation. Achieving high yield requires systematically optimizing the potential of both the source and sink for high-level coordination. Therefore, evaluating the characteristics of source and sink strength is critical for understanding the physiological role of source-sink theory in crop yield regulation.

In recent years, significant research has been devoted to elucidating the physiological mechanisms of the source-sink relationship. However, due to the genetic background of the tested cultivars, previous studies have struggled to accurately define the functional relationship between source capacity and sink strength at the cultivar level. To gain a better understanding of how source-sink relationships ultimately impact wheat yield, this study selected three wheat cultivars and applied two N fertilizer levels to compare the source-sink characteristics and types. While our previous work explored photosynthetic and photoprotective mechanisms in these cultivars (Zhang et al., 2024a, 2025a), the present study focuses specifically on establishing a systematic framework for source-sink characterization and classification, providing the physiological basis for understanding yield limitation types. It holds significant implications for optimizing high-yield, high-quality wheat production.

## 2 Material and methods

### 2.1 Experiment design

**Figure 1:**
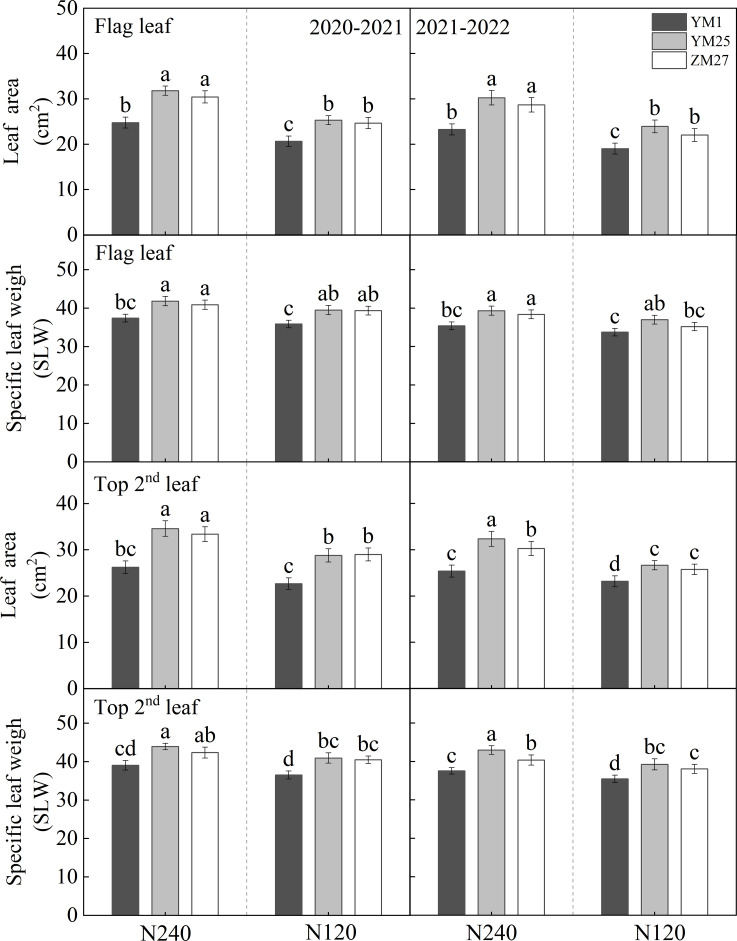
Differences in leaf area and specific leaf weight (SLW) at anthesis in different wheat cultivars. N240 and N120 respectively represent nitrogen application rates of 240 and 120 kg^·^hm^-2^. Different lowercase letters above bars indicate statistically significant differences between treatments (*p* < 0.05) according to the LSD test. Error bars represent standard deviation (SD) of three biological replicates.

The experiment was conducted at the experimental base of Nanjing Agricultural University (32°24’N, 118°9’E) in Nanjing, China, during the 2020/2021 and 2021/2022 growing seasons. A field experiment was conducted, selecting three different wheat cultivars: Yangmai 1 (YM1, southern cultivar, sink-limited wheat), Yangmai 25 (YM25, southern cultivar, source-limited wheat), and Zhoumai 27 (ZM27, northern cultivar, source-limited wheat), which were studied in our previous work (Zhang et al., 2024a, 2025a). According to the response of different cultivars to source and sink manipulations, YM25 and ZM27 had a higher sink-source ratio, manifesting as a source-limited cultivar. YM1 has a lower sink-source ratio, manifesting as a sink-limited cultivar. Two N fertilizer treatments were set, with N application rates of 240 kg·hm^-2^ and 120 kg·hm^-2^, referred to as N240 and N120, respectively. A completely randomized block design was used, with N fertilizer as the main factor and cultivar as the secondary factor. The basic seeding was 2.25×10^6^ seeds hm^-2^. The plot size was 3.0 m×3.0 m (9m^2^) with 12 rows and a spacing of 0.25 m between rows. 50% of the N fertilizer and all phosphorus (P_2_O_5_, 150 kg·hm^-2^) and potassium fertilizer (K_2_O, 150 kg·hm^-2^) were used as base fertilizer, with the remaining 50% of N applied at the jointing stage. Soil samples at a depth of 0 to 25 cm were found to have 20.44 g·kg^–1^ organic matter, 1.35 g·kg^–1^ total N content, 65.22 mg·kg^–1^ available N content, 18.49 mg·kg^–1^ available P_2_O_5_ content, and 109.44 mg·kg^–1^ rapidly available K in 2020–2021 and 26.98 g·kg^–1^ organic matter, 1.40 g·kg^–1^ total N content, 68.22 mg·kg^–1^ available N content, 12.37 mg·kg^–1^ available P_2_O_5_ content, and 93.76 mg·kg^–1^ rapidly available K in 2021-2022. The sowing dates were November 2 in 2020, and November 13 in 2021, and the harvesting dates were May 24, 2021, and May 26, 2022. Seedlings were thinned at the three-leaf stage, and stakes were provided to prevent lodging for lodging-prone cultivars YM1, ensuring stable yield performance for all cultivars. Management followed local cultivation practices, with timely pest and disease control. The average temperature and cumulative precipitation meteorological conditions during wheat growth were calculated according to the meteorological data provided by the local weather station and are shown in **Figure 1**. The climate is humid and warm, with an annual rainfall of 476.60 mm and 568.76 mm during 2020–2021 and 2021-2022, respectively. The range of monthly average temperatures in the region was 4-22 °C and 4-22 °C during 2020–2021 and 2021-2022, respectively. Meteorological conditions during the wheat growing seasons were typical for the region, with no extreme events noted that would significantly impact the treatments differently.

YM1 is a spring wheat cultivar with a full growth period of approximately 204 days. The seedlings exhibit semi-prostrate growth, strong tillering ability, and a high ear-setting rate. The plant type is loose, with short and narrow leaves. The plant height reaches 100 cm, exhibiting poor lodging resistance and an average maturity appearance. It has a high N fertilizer requirement. YM25, also a spring wheat cultivar, has a full growth period of 202 days. The seedlings are semi-prostrate, exhibit strong tillering ability, and have a high ear-setting rate. The plant type is compact, with upright leaves and a relatively neat ear arrangement. The plant height is 83 cm, showing good lodging resistance and an excellent maturity appearance. It has a moderate N fertilizer requirement. ZM27, a semi-winter wheat cultivar, has a full growth period of 208 days. The seedlings exhibit semi-prostrate growth, moderate tillering ability, and a medium ear-setting rate. The plant type is somewhat loose, with narrow and elongated leaves. The flag leaf is long and curls upwards. The plant height is 74 cm, showing moderate lodging resistance and an average maturity appearance. It has a medium N fertilizer requirement.

### 2.2 Yield and yield components

Grain yield and its components were recorded at maturity for all treatments. Specifically, plants were harvested from each subplot in a 1-meter double-row, manually threshed, naturally sun-dried, and weighed to obtain yield, 1000-kernel weight (TKW) (adjusted to 13% moisture), and the number of ears per square meter. Additionally, 30 ears were collected from each subplot to measure the number of grains per ear.

### 2.3 Leaf area, specific leaf weight, and leaf area index

Twenty single stems were collected at the jointing, booting, anthesis, and grain filling stages. Leaf area and LAI were calculated using the SLW method.


$$\text{Leaf area}=\text{leaf sample area}({\text{cm}}^{2})\times \text{leaf dry weight}(\text{mg})/\text{sample dry weight}(\text{mg});$$

$$\text{Leaf area index}(\text{LAI},{\text{m}}^{2}/{\text{m}}^{2})=\text{single stem leaf area}({\text{cm}}^{2})\times \text{number of ears per square meter}/1{\text{m}}^{2}$$

### 2.4 Measurement of SPAD duration

Single stems, which flowered on the same day and were uniform in size, were tagged at anthesis. SPAD values of the tagged flag leaves were measured at -10, 0, 10, 20, and 30 days after anthesis. Chlorophyll SPAD values of wheat flag leaves were directly measured in the field using a SPAD-502 chlorophyll meter (Konica Minolta Holdings, Inc., Japan). In each subplot, six leaves were randomly selected, with measurements taken at the upper, middle, and lower sections of each leaf. Each section was measured three times to calculate the average value. The SPAD duration (SAD) was calculated using the method described by Tian et al. (2011), as shown in Equation 1.

(1) $$\text{SAD}=\int_{0}^{\text{D}}\text{f}(\text{x})\text{dx}-\text{SD}\times \frac{\text{SPADmax}}{2}$$

f(x) represents the SPAD fitting equation, SD refers to the SPAD duration, which is the time (in days) that SPAD stays at half of the maximum SPAD value (SPAD_max_), and SPAD_max_ is the maximum SPAD value, the peak of the curve f(x).

### 2.5 Measurement of photosynthetic duration

The net photosynthetic rate (Pn) of flag leaves was measured at -10, 0, 10, 20, and 30 days after anthesis (DAA) using a gas exchange system (LI-6400, LI-Cor Inc., Lincoln, NE, USA) during 09:00-11:00 a.m. on clear days without clouds. The environmental conditions in the leaf chamber were according to Mu and Chen, 2021. The photosynthetic active radiation (PAR) was 1200 μmol·m^-2^·s^-1^, the leaf chamber CO_2_ concentration was about 400 ± 20 μmol·CO_2_·L^-1^, the leaf temperature was 25.0 ± 0.5°C, and the humidity was 60%. Three leaves of uniform growth were selected from each plot, and data were recorded three times after a 10-minute light induction period when the photosynthetic value stabilized. The photosynthetic duration (PAD) was calculated using the method described by Liu et al. (2020), as shown in Equation 2.

(2) $$\text{PAD}=\int_{0}^{\text{D}}\text{f}(\text{x})\text{dx}-\text{PD}\times \frac{\text{Pnmax}}{2}$$

f(x) represents the photosynthetic rate fitting equation, PD refers to the photosynthetic duration, which is the time (in days) that the photosynthetic rate remains at half of the maximum photosynthetic rate (Pn_max_), and Pn_max_ is maximum Pn value, the peak value of the curve f(x).

### 2.6 Measurement of spike and grain traits

At maturity, uniform and healthy plants were selected to collect 20 single spikes. Spike length, number of spikelets, and single spike yield were measured. Grains were harvested, and their volume was measured using the displacement method, and the grain weight was recorded. The number of kernels per spikelet and grain filling rate were calculated using the following formulas:

Number of kernels per spikelet=Total grains/Fertile spikelets

Grain density=Total grains/Spike length


Grain filling rate=Grain weight/Grain volume

### 2.7 Measurement of grain filling rate and duration of grain filling

At anthesis, single stems with uniform anthesis date and size were tagged. Sampling was carried out from the marked plants at 5, 10, 15, 20, 25, 30, 35 days after anthesis, and at maturity. For each sample, 20 spikes were harvested, manually threshed, and placed in an oven at 105 °C for 30 minutes for heat shock, followed by drying at 70 °C until a constant weight was achieved. The TKW was then measured. The grain filling process of wheat was fitted using the Richards growth regression equation (Richards, 1959) to calculate the grain filling rate and key parameters.

(3) $$\text{W}=\frac{\text{A}}{{\left(1+{\text{Be}}^{-\text{kt}}\right)}^{1/\text{N}}}$$

1000-kernel weight(W) was shown in Equations 3. The grain filling rate (R) was calculated using the first derivative, as shown in Equation 4.

(4) $$\text{R}=\frac{\left({\text{AKBe}}^{-\text{kt}}\right)}{\text{N}{\left(1+{\text{Be}}^{-\text{kt}}\right)}^{(\text{N}+1)/\text{N}}}$$

In the equation, B, K, and N represent the regression coefficients, W is the 1000-kernel weight (mg), A is the final 1000-kernel weight (mg), and t represents the number of days for grain filling (days).

### 2.8 Source-sink ratio

The source-sink ratio can comprehensively evaluate the relationship between the source and sink from both perspectives. The source-sink ratio is calculated as follows (Zhang et al., 2025b)


Source−sink ratio=Single spike yield/Maximum single stem leaf area at anthesis.

### 2.9 Statistical analysis

Data were analyzed using Excel 2019 and SPSS Statistics 24 (SPSS Inc., Chicago, IL, USA), followed by analysis of variance (ANOVA), significance tests, and correlation analysis. Mean comparisons were performed using the Least Significant Difference (LSD) method, with all significance analyses conducted at the *p* < 0.05 level. All figures and tables were generated using Origin 2021 (Systat Software, USA). Correlation and variance analyses were conducted using the three-year average data.

## 3 Results

### 3.1 Leaf area and specific leaf weight

Under both N240 and N120, the top three leaves of YM25 and ZM27 at anthesis exhibited significantly larger leaf area and higher SLW compared to YM1 (**Figure 1**; **Supplementary Table 1**). N fertilizer played a crucial role in regulating these source traits. Under low-N conditions, all cultivars showed marked reductions in the area and SLW of their upper leaves. These results suggest that YM25 and ZM27 enhanced their source strength through increased leaf area, greater leaf thickness, and improved SLW. In contrast, YM1 had smaller and thinner leaves with lower SLW, indicating insufficient source activity. The nutrient deficiency under low-N conditions further suppressed leaf development and source activity, ultimately leading to reduced photosynthetic capacity. YM25 and ZM27 responded more robustly to N increments compared to YM1.

### 3.2 Leaf area index

**Figure 2:**
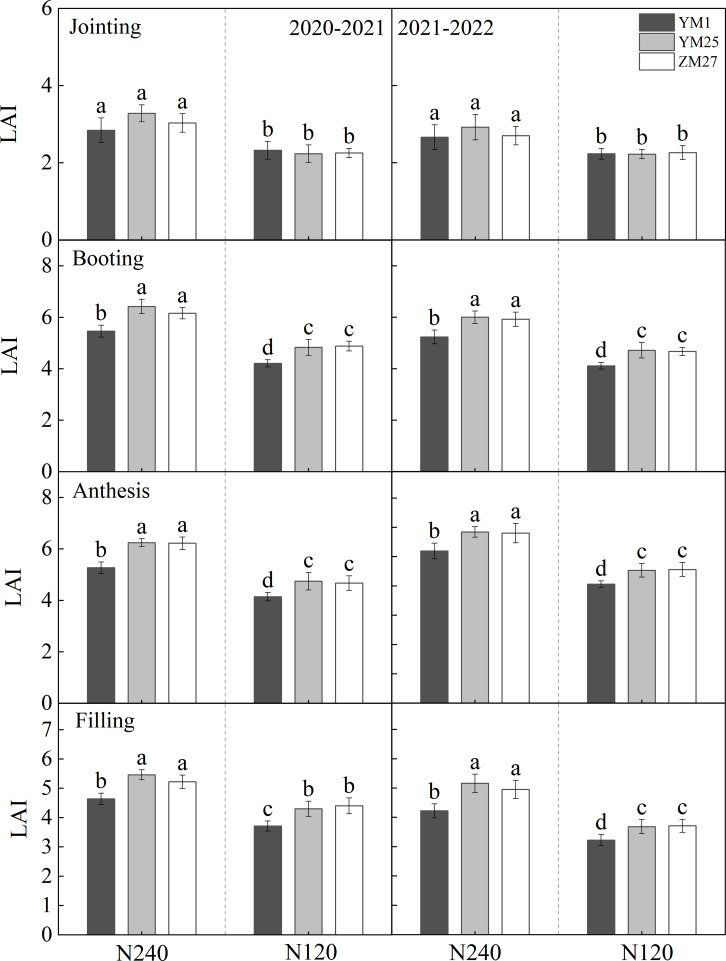
Differences in LAI in different wheat cultivars. N240 and N120 respectively represent nitrogen application rates of 240 and 120 kg^·^hm^-2^. Different lowercase letters above bars indicate statistically significant differences between treatments (*p* < 0.05) according to the LSD test. Error bars represent standard deviation (SD) of three biological replicates.

The LAI of all wheat cultivars first increased and then decreased with developmental progression, peaking at the booting stage (**Figure 2**; **Supplementary Table 2**). Under both N240 and N120, YM25 and ZM27 exhibited significantly higher LAI than YM1 during booting, anthesis, and grain-filling stages, although no significant difference was observed at the jointing stage. Low N conditions markedly reduced LAI across all cultivars. These results indicate that YM25 and ZM27, in comparison with YM1, were able to maintain higher LAI values, resulting in enhanced canopy light interception. This improvement likely prolonged the functional lifespan of leaves and delayed the senescence of photosynthetically active tissues. However, under N-deficient conditions, the decline in LAI due to limited nutrient availability reduced the light interception area at the canopy level and weakened photosynthetic efficiency.

### 3.3 Duration of SPAD

The SPAD value of the flag leaf followed a quadratic trend throughout the growth period (**Table 1**). The fitted equations demonstrated statistically significant correlation coefficients. Under both N240 and N120, YM25 and ZM27 exhibited significantly higher SPAD_max_ and longer SAD compared to YM1. Low N conditions markedly reduced these parameters in all cultivars. These results suggest that the enhanced SPAD_max_ and extended SPAD duration in YM25 and ZM27 provide a physiological foundation for sustaining high photosynthetic activity after anthesis. In contrast, YM1 displayed more rapid leaf senescence, with a lower maximum SPAD_max_ and shorter SAD, which limited its capacity for light absorption and utilization.

**Table 1:** Differences in SPAD duration of flag leaves in different wheat cultivars.

Cultivars	N rates	Fitting equation	R^2^	SPAD_max_	SAD (SPAD·d)
2020-2021					
YM1	N240	Y = 48.960-0.111 X-0.021 X^2^	0.999**	49.11bc	494.13c
	N120	Y = 41.766-0.066 X-0.020 X^2^	0.993**	41.82d	415.20d
YM25	N240	Y = 55.180-0.022 X-0.022 X^2^	0.995**	55.19a	635.02a
	N120	Y = 47.002-0.106 X-0.021 X^2^	0.999**	47.14c	466.18c
ZM27	N240	Y = 52.829 + 0.006 X-0.024 X^2^	0.995**	52.83ab	584.58b
	N120	Y = 46.584-0.081 X-0.023 X^2^	0.993**	46.66cd	453.14cd
	F-value			9.13**	36.64**
2021-2022					
YM1	N240	Y = 46.151-0.142 X-0.018 X^2^	0.995**	46.43b	301.83e
	N120	Y = 39.222-0.185 X-0.014 X^2^	0.988**	39.83c	370.56d
YM25	N240	Y = 52.119-0.102 X-0.018 X^2^	0.994**	52.26a	588.84a
	N120	Y = 44.369-0.066 X-0.021 X^2^	0.996**	44.42bc	305.63e
ZM27	N240	Y = 50.643-0.188 X-0.016 X^2^	0.983**	51.21a	533.20b
	N120	Y = 42.319-0.127 X-0.018 X^2^	0.993**	42.54bc	412.33c
	F-value			10.71**	100.06**

N240 and N120 respectively represent nitrogen application rates of 240 and 120 kg·hm^-2^. Values are means of three replicates. F-values were calculated by two-way ANOVA, ** represents *p* < 0.01, and different letters in a column indicate significant differences (*p* < 0.05), according to the LSD test. SPAD_max_, Max SPAD; SAD, SPAD duration.

### 3.4 Photosynthetic duration

The photosynthetic rate of the flag leaf exhibited a quadratic trend throughout the developmental stages, and the fitted equations had statistically significant correlation coefficients (**Table 2**). Under both N240 and N120, YM25 and ZM27 showed significantly higher Pn_max_ and longer PAD than YM1, with no significant differences observed between YM25 and ZM27. In contrast, low-N conditions led to a marked reduction in both Pn_max_ and PAD across all cultivars. These results indicate that YM25 and ZM27 achieved enhanced photosynthetic performance by increasing Pn_max_ and extending the duration of photosynthesis, thereby improving post-anthesis photosynthetic capacity. On the other hand, YM1 exhibited faster senescence of source leaves, lower Pn_max_, and shorter PAD, ultimately resulting in insufficient post-anthesis carbon assimilation.

**Table 2:** Differences in photosynthesis duration in flag leaves of different wheat cultivars.

Cultivars	N rates	Fitting equation	R^2^	Pn_max_ (μmol CO_2_·m^-2^·s^-1^)	PAD (μmol·CO_2_·m^-2^·s-^1^·d)
2020-2021					
YM1	N240	Y = 21.104-0.058 X-0.012 X^2^	0.994**	21.17b	183.83c
	N120	Y = 16.673-0.063 X-0.010 X^2^	0.988**	16.77d	135.48e
YM25	N240	Y = 24.518-0.000 X-0.013 X^2^	0.986**	24.52a	249.76a
	N120	Y = 19.835-0.019 X-0.013 X^2^	0.996**	19.84bc	174.83c
ZM27	N240	Y = 22.920-0.038 X-0.012 X^2^	0.996**	22.95a	217.85b
	N120	Y = 18.667-0.063 X-0.012 X^2^	0.997**	18.75c	149.98d
	F-value			26.21**	72.18**
2021-2022					
YM1	N240	Y = 18.672-0.064 X-0.010 X^2^	0.997**	18.77b	161.67c
	N120	Y = 15.279-0.081 X-0.008 X^2^	0.975**	15.48c	121.00e
YM25	N240	Y = 22.493-0.037 X-0.011 X^2^	0.966**	22.52a	220.75a
	N120	Y = 18.231-0.052 X-0.010 X^2^	0.983**	18.30b	160.54c
ZM27	N240	Y = 21.013-0.059 X-0.011 X^2^	0.994**	21.09a	189.22b
	N120	Y = 17.188-0.025 X-0.012 X^2^	0.973**	17.20b	144.44d
	F-value			25.88**	60.57**

N240 and N120 respectively represent nitrogen application rates of 240 and 120 kg·hm^-2^. Values are means of three replicates. F-values were calculated by two-way ANOVA, ** represents *p* < 0.01, and different letters in a column indicate significant differences (*p* < 0.05), according to the LSD test. Pn_max_, Max net photosynthetic rate; PAD, Photosynthetic duration.

### 3.5 Grain yield and its components

**Figure 3:**
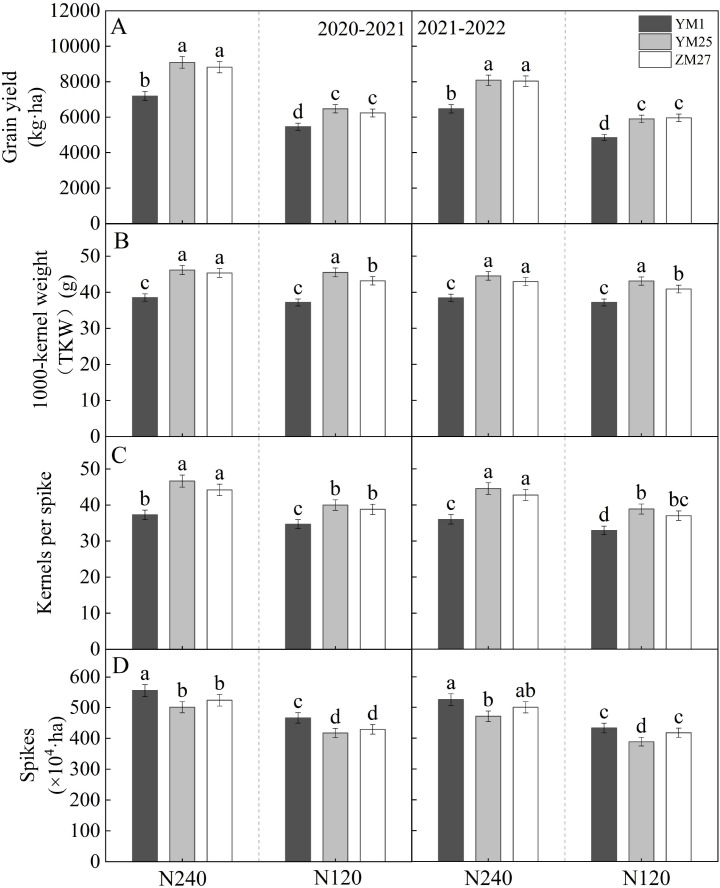
Comparison of grain yield **(A)**, 1000-kernel weight **(B)**, kernels per spike **(C)**, and spikes **(D)** of wheat in different wheat cultivars. N240 and N120 respectively represent nitrogen application rates of 240 and 120 kg^·^hm^-2^. Different lowercase letters above bars indicate statistically significant differences between treatments (*p* < 0.05) according to the LSD test. Error bars represent standard deviation (SD) of three biological replicates.

Significant differences were observed among wheat cultivars in terms of grain yield, TKW, kernels per spike, and spikes, (**Figures 3A–D**). Under both N240 and N120, YM25 and ZM27 exhibited significantly higher grain yield, kernels per spike, and TKW compared to YM1, despite having fewer spikes. Specifically, YM25 had fewer spikes than ZM27 but more kernels per spike, and their TKW were not significantly different, resulting in no significant difference in final yield between the two. Low N conditions significantly reduced grain yield, spikes, and kernels per spike in all cultivars, whereas TKW remained unaffected. These results indicate that the yield advantages of YM25 and ZM27 were primarily driven by increases in kernels per spike and TKW rather than spikes. In contrast, YM1 exhibited higher spikes but produced lower yield due to reduced kernels per spike and TKW, which can be attributed to differences in source-sink relationship among the cultivars.

### 3.6 Spike traits

**Figure 4:**
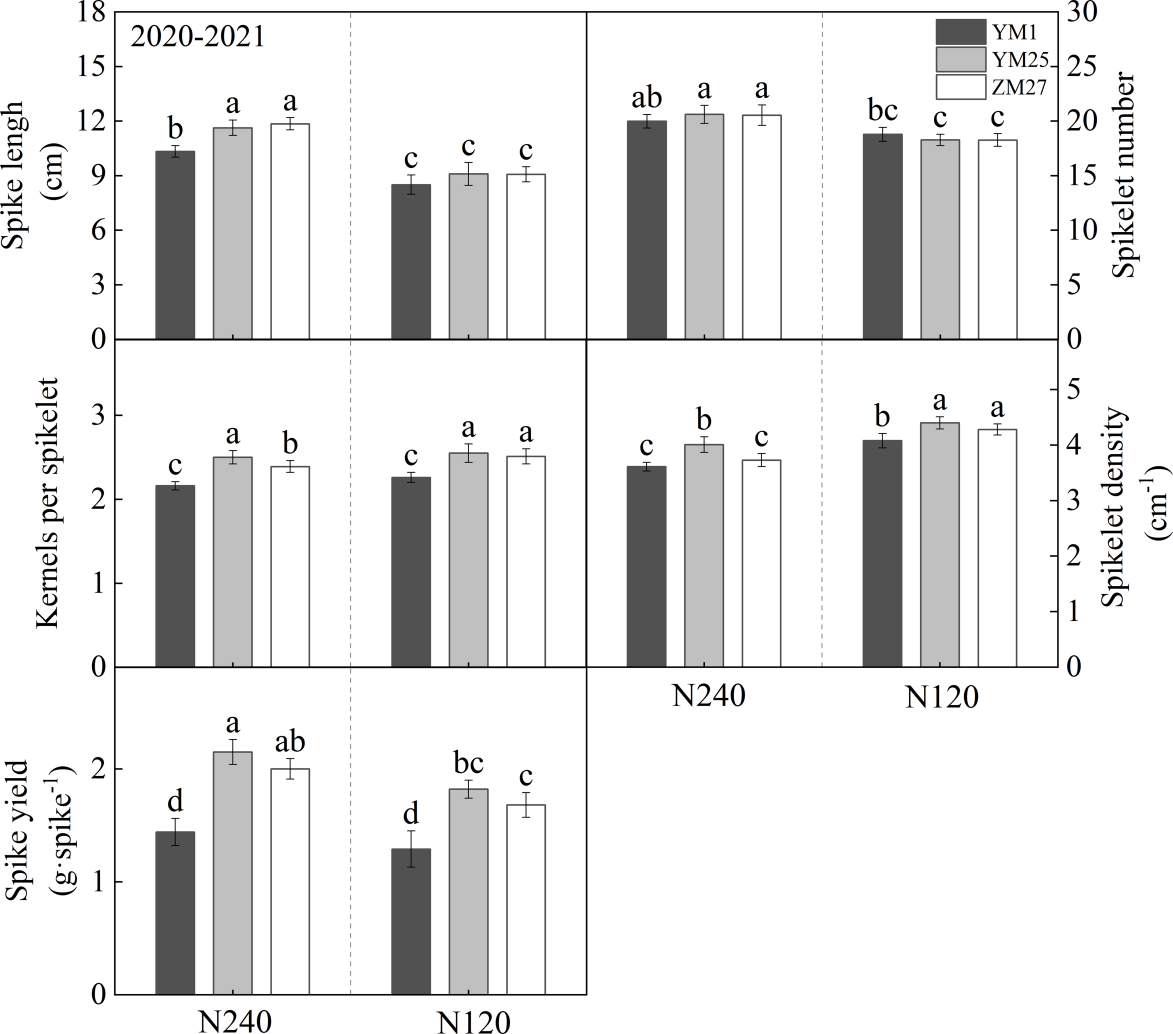
The differences of spike characterisrics in different wheat cultivars. N240 and N120 respectively represent nitrogen application rates of 240 and 120 kg^·^hm^-2^. Different lowercase letters above bars indicate statistically significant differences between treatments (*p* < 0.05) according to the LSD test. Error bars represent standard deviation (SD) of three biological replicates.

There were distinct differences in spike-related traits among wheat cultivars (**Figure 4****;** **Supplementary Table 3**). Under both N240 and N120, YM25 and ZM27 exhibited significantly greater spike length, kernels per spikelet, spikelet density, and spike yield compared to YM1, while no significant differences in spikelet number were observed among the cultivars. N application had a strong regulatory effect on spike development. Under low-N conditions, all cultivars showed a significant decrease in spikelet number and spike yield, but an increase in kernels per spikelet and spikelet density. These findings indicate that the improvement in spike grain number in YM25 and ZM27, relative to YM1, was primarily driven by increased kernels per spikelet rather than changes in spikelet number. YM25 and ZM27 responded more robustly to N increments compared to YM1.

### 3.7 Grain traits

**Figure 5:**
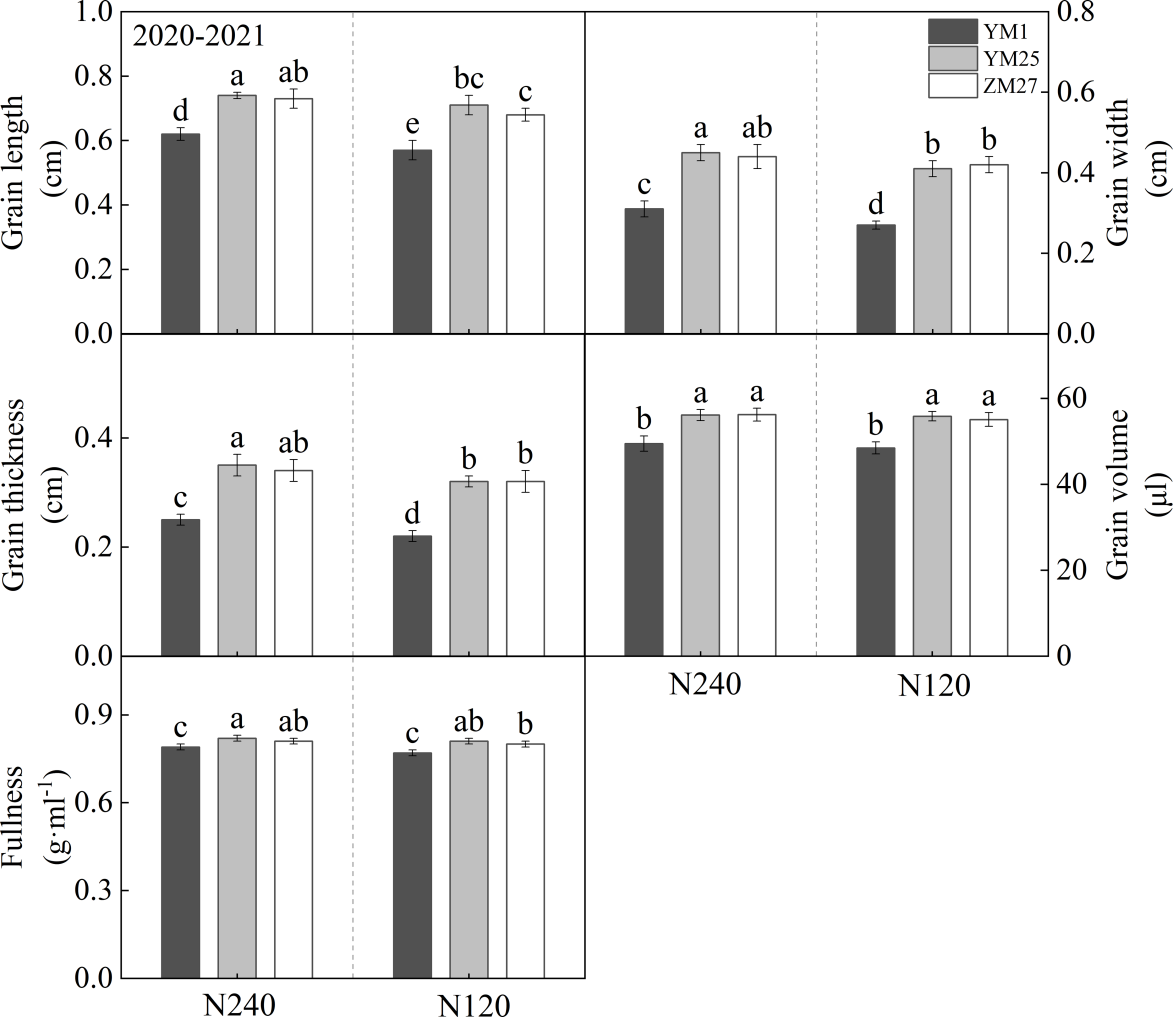
The differences of grain characteristics in different wheat cultivars. N240 and N120 respectively represent nitrogen application rates of 240 and 120 kg^·^hm^-2^. Different lowercase letters above bars indicate statistically significant differences between treatments (*p* < 0.05) according to the LSD test. Error bars represent standard deviation (SD) of three biological replicates.

Notable differences in grain traits were observed among wheat cultivars (**Figure 5**; **Supplementary Table 4**). Under both N240 and N120, YM25 and ZM27 demonstrated significantly greater grain length, grain thickness, grain volume, and higher grain fullness than YM1. N application had a strong regulatory effect on grain development, whereas N deficiency significantly reduced grain volume and grain fullness across all cultivars. These results indicate that YM25 and ZM27 formed plumper, better grain fullness, suggesting enhanced sink capacity. In contrast, YM1 produced smaller and lighter grains with a lower grain fullness, reflecting a limited sink potential. YM25 and ZM27 responded more robustly to N increments compared to YM1.

### 3.8 Grain filling characteristics

Grain filling duration and rate are critical determinants of final grain weight, and were modeled using a logistic growth equation. All fitted logistic models achieved highly significant correlation coefficients (**Table 3**). Under both N240 and N120, YM25 and ZM27 demonstrated significantly higher G_max_ and G_mean_ than YM1, accompanied by prolonged final grain filling duration (T_final_) and earlier peak filling periods (D_active_). In contrast, under low-N conditions, all cultivars experienced a marked reduction in both T_final_ and D_active_ (2021-2022), although no significant differences were observed in G_max_ and G_mean_. These results suggest that YM25 and ZM27 had higher G_max_ and G_mean_, with the peak filling period (D_active_) occurring much earlier, extended filling duration (T_final_), and higher final grain weight. YM1, however, exhibited a lower G_max_, shorter T_final_, lower grain fullness, and lower final grain weight.

**Table 3:** Differences in grain filling parameters in different wheat cultivars.

Cultivars	N rates					R^2^	T_final_ (d)	D_active_ (d)	G_max_ (mg·d^-1^)	G_mean_ (mg·d^-1^)
		A	B	K	N					
2020-2021										
YM1	N240	37.40	14.45	0.21	0.60	0.993**	36.68b	18.72a	1.69cd	1.09de
	N120	34.35	20.27	0.22	0.66	0.993**	32.69c	18.03ab	1.61d	0.99e
YM25	N240	45.38	1.63	0.14	0.21	0.975**	38.81a	17.14bc	2.00a	1.37a
	N120	41.58	1.77	0.14	0.22	0.976**	35.70b	16.97c	1.86ab	1.25bc
ZM27	N240	42.87	3.06	0.16	0.30	0.985**	39.65a	17.59bc	1.86ab	1.28ab
	N120	38.90	3.21	0.16	0.31	0.986**	36.90b	17.54bc	1.78bc	1.16cd
F-value							19.13**	5.57**	8.13**	19.23**
2021-2022										
YM1	N240	37.06	3.78	0.17	0.33	0.992**	37.22c	19.20a	1.74bc	1.09cd
	N120	35.16	7.00	0.19	0.45	0.996**	33.70d	17.83b	1.67c	1.02d
YM25	N240	44.18	2.06	0.15	0.24	0.979**	39.01ab	18.29b	1.96a	1.33a
	N120	41.62	4.07	0.17	0.36	0.991**	36.83c	16.84c	1.86ab	1.23ab
ZM27	N240	41.53	1.98	0.15	0.24	0.98**	40.29a	18.25b	1.87ab	1.25a
	N120	38.82	3.28	0.16	0.31	0.989**	37.37bc	16.89c	1.77bc	1.15bc
F-value							16.99**	11.07**	4.68*	13.03**

N240 and N120 respectively represent nitrogen application rates of 240 and 120 kg·hm^-2^. Values are means of three replicates. F-values were calculated by two-way ANOVA, * represents *p* < 0.05, ** represents *p* < 0.01, and different letters in a column indicate significant differences (*p* < 0.05), according to the LSD test. A, final 1000-kernel weight; B, K, N, regression coefficients from the Richards growth equatio; T_final_, Final grain filling duration; D_active_, Active grain filling period; G_max_, Maximum filling rate; G_mean_, Mean filling rate; n.

### 3.9 Sink-source ratio

**Figure 6:**
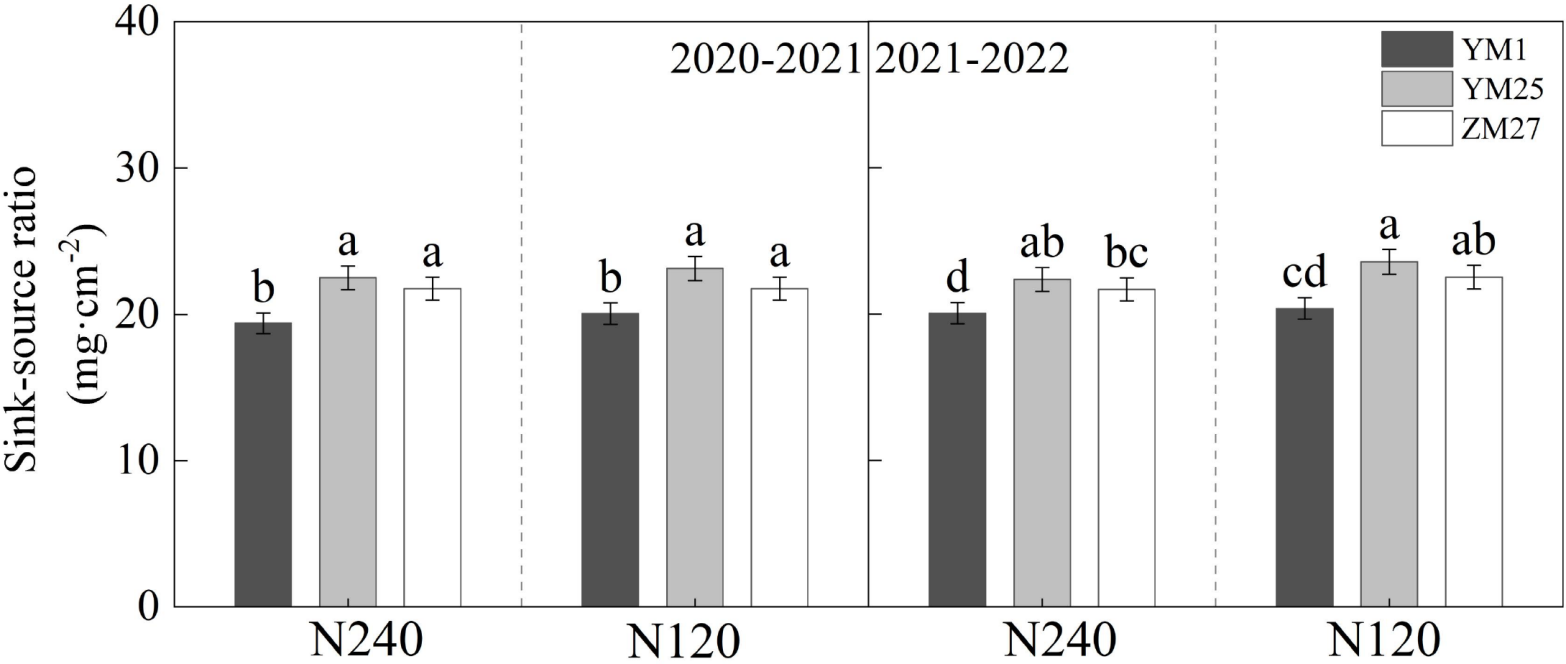
Differences of sink-source ratio in different wheat cultivars.N240 and N120 respectively represent nitrogen application rates of 240 and 120 kg^·^hm^-2^. Different lowercase letters above bars indicate statistically significant differences between treatments (*p* < 0.05) according to the LSD test. Error bars represent standard deviation (SD) of three biological replicates.

YM25 and ZM27 exhibited significantly higher sink-source ratios than YM1, indicating that the increase in per-spike yield in these modern cultivars outpaced the increase in flag leaf area at anthesis (**Figure 6**). This suggests that the yield formation in YM25 and ZM27 is increasingly constrained by source capacity. Both cultivars showed a pattern of relatively small source and large sink, implying that insufficient assimilate supply from the photosynthetic apparatus is the main limiting factor for further yield improvement. In contrast, YM1 exhibited the opposite trend—stronger source capacity but limited sink development—suggesting that sink size restricts its yield potential. Moreover, N application played a key regulatory role in the source-sink balance. With increasing N levels, the sink-source ratio decreased, indicating that N promoted leaf area expansion more than per-spike yield, thus effectively mitigating source limitation.

### 3.10 Correlation analysis

**Figure 7:**
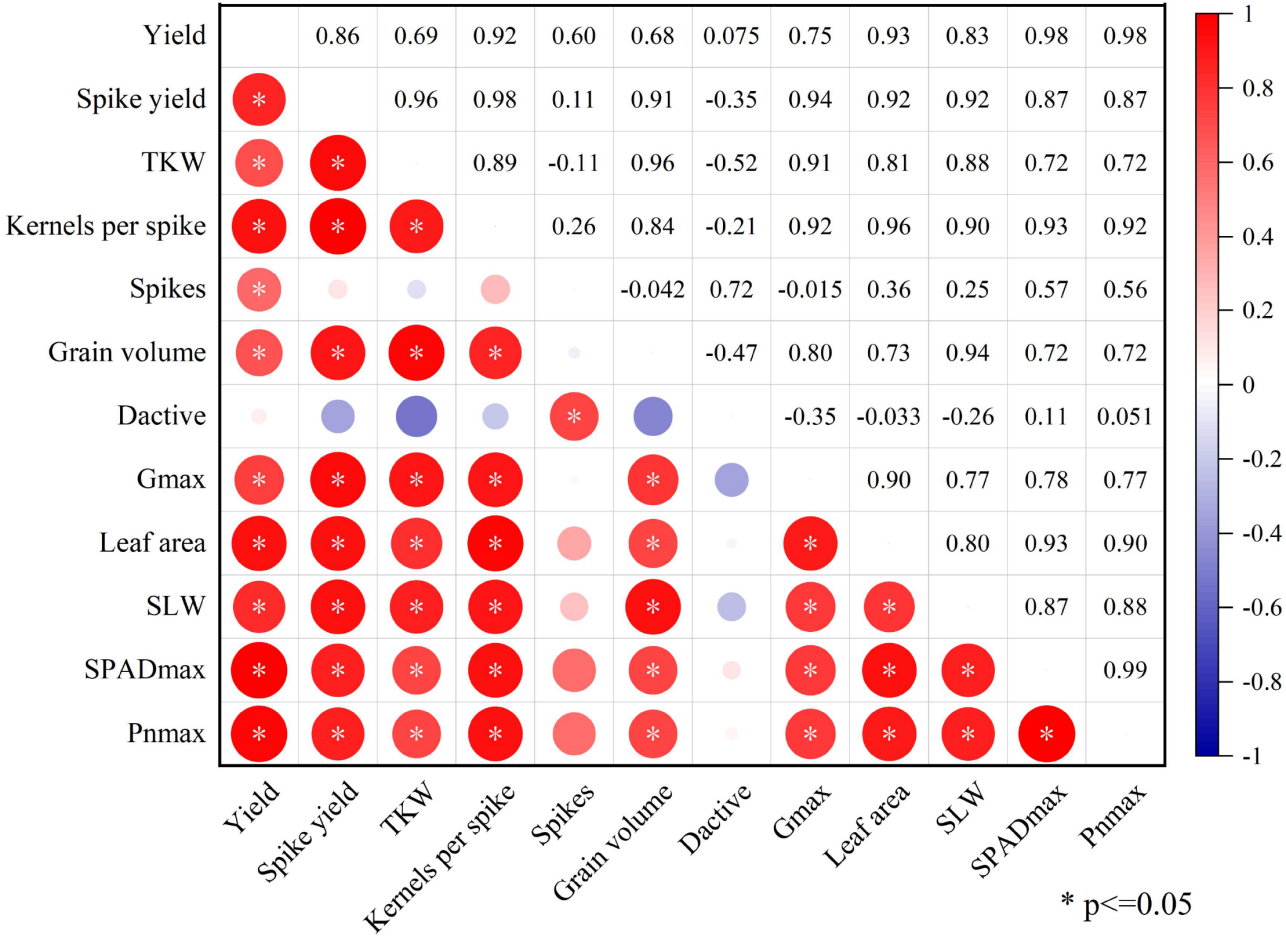
Correlation analysis among indexes of source-sink traits. The figure shows the correlation analysis between various indicators of traits in the data source database from 2020 to 2022. * represents *p* < 0.05,. Yield, Grain yield; Spike yield, yield per spike; TKW, 1000-kernel weight; Kernels per spike, Kernels per spike; Spikes, Spikes; Grain volume, Grain volume; D_active_, Active grain filling period; G_max_, Maximum filling rate; Leaf area, Leaf area; SLW, Specific leaf weight; SPAD_max_, Max SPAD; Pn_max_, Max net photosynthetic rate.

Grain yield exhibited significant positive correlations with a range of agronomic and physiological traits, including spike yield, TKW, Kernels per spike, spikes, grain volume, G_max_, leaf area, SLW, SPAD_max_, and Pn_max_ (**Figure 7**). These relationships indicate that improvements in source-sink traits are closely linked to increased grain yield in wheat. Notably, both leaf area and SLW were significantly and positively correlated with key sink traits such as spike yield, TKW, grain volume, and G_max_. This suggests that enhancing the assimilate supply capacity of the source is essential for expanding grain sink size and improving sink activity.

## 4 Discussion

### 4.1 Physiological mechanisms of yield formation in different wheat cultivars

The formation of grain yield is a synergistic process involving the accumulation and distribution of photosynthates (Gao et al., 2017; Zhang et al., 2024b). In this study, significant yield differences were observed among the three cultivars (**Figure 3A**). YM25 and ZM27 exhibited a substantial yield advantage over YM1, which was primarily attributed to their superior sink capacity, specifically higher TKW and kernel per spike, but spikes showed a decreasing trend (**Figures 3B, C**). This suggests that the increase in yield of YM25 and ZM27 is mainly attributed to the increase in kernel per spike and grain weight. This is consistent with the findings of Reynolds et al. (2009), who emphasized that expanding sink size is a prerequisite for achieving high yield potential. To achieve high crop yield, it is necessary to combine plant architecture with the population, thus coordinating the source-sink relationship and maximizing the conversion of photosynthetic products into grains.

In current research, the main indicators used to measure source traits include the net photosynthetic rate, carboxylation efficiency, chlorophyll content, and chlorophyll fluorescence parameters, which describe source activity, as well as leaf area and LAI, which describe source size (Ma et al., 2025; Tan et al., 2025; Wang and Dang, 2024). Our study shows that the robust sink capacity of YM25 and ZM27 was fundamentally supported by their enhanced source strength at anthesis. High-yielding cultivars possessed significantly larger leaf areas, higher SLW, and LAI in the top three functional leaves (**Figures 1**, **2**). As suggested by Fabre et al. (2020), a higher SLW often indicates a higher concentration of photosynthetic enzymes and thicker mesophyll, contributing to a stronger “potential source.” Moreover, the stay-green characteristics observed in YM25 and ZM27, characterized by slower SPAD_max_ and higher SPAD duration (**Table 1**), and the Pn_max_ is higher, with a prolonged photosynthetic duration (**Table 2**). These provided a stable supply of assimilates during the critical grain-filling period, thereby preventing premature senescence-induced yield loss (Zhang et al., 2016). In contrast, YM1 has insufficient leaf-source, with smaller SLW, leaf area, and LAI, lower SPAD_max_ and Pn_max_, weakened post-anthesis photosynthetic capacity, and inadequate source ability (**Figures 1**, **2**; **Tables 1**, **2**).

### 4.2 Grain filling dynamics and assimilate partitioning

The grain-filling process is a critical determinant of grain weight and final yield. In current research, indicators used to measure sink traits include sink capacity (TKW, kernels per spike), sink strength, and duration of grain filling (Serrago et al., 2025). Compared with YM1, the increase in kernels per spike in YM25 and ZM27 is mainly due to the increase in the number of kernels per spikelet, rather than spikelet number (**Figure 4**). The increase in TKW is mainly attributed to the simultaneous increase in grain weight, grain volume, and grain fullness (**Figure 5**). Wheat grain filling characteristics can be categorized -–-into filling rate and filling duration, and both improving the filling rate and extending the filling duration can enhance dry matter accumulation in grains. This study shows that YM25 and ZM27 have higher G_max_ and G_mean_ during the filling period, with the peak filling period occurring much earlier, extended filling duration, increased grain fullness, and higher final grain weight (**Table 3**). This “early-and-fast” filling pattern is essential for maximizing TKW, especially under environmental conditions where late-season heat or drought might induce premature senescence. In contrast, YM1 exhibited a lower filling rate and reduced grain fullness (**Table 3**), which may be attributed to its limited source supply (as evidenced by its smaller leaf area) and lower sink activity. The significant correlation between filling rate and final grain weight across N treatments further underscores the importance of filling intensity in yield formation.

### 4.3 Cultivar classification based on source-sink relationships

Wheat grain yield is determined by the synergistic interaction of source activity and sink capacity. Directly determining the degree of source or sink limitation in crop growth is crucial for improving yield (Burnett et al., 2021). A central objective of this study was to classify wheat cultivars based on their limiting factors. Based on the source-sink ratio and yield components, we classified YM25 and ZM27 as source-limited types, while YM1 was identified as a sink-limited type. In YM25 and ZM27, although absolute source strength was high, the exceptionally large sink capacity (high kernels per spike and potential grain size) resulted in a high sink-source ratio (**Figure 6**). In these cultivars, the “sink” demand appears to outpace the “source” supply, making further yield increases dependent on enhancing leaf photosynthetic efficiency or extending functional duration. Conversely, YM1 represents a low-yield equilibrium characterized by both a weak source and a weak sink. Its lower sink-source ratio suggests that even with its limited source, the sink is insufficient to utilize all available assimilates, indicating that increasing sink size should be the priority for this type of cultivar (Zhang et al., 2010). Source-limited cultivars of YM25 and ZM27 are relative. While they possess a higher absolute source capacity (leaf area and SLW) than YM1, their massive sink potential (grain number and volume) creates a higher demand that current photosynthesis alone cannot fully satisfy. Additional N fertilization alleviates this relative limitation by extending leaf senescence (SPAD duration), thereby narrowing the gap between source supply and sink demand. For sink-limited cultivars like YM1, since their source capacity (leaf area and SLW) is relatively sufficient for their smaller sink, the priority should be placed on increasing the number of spikes per unit area or kernels per spike through early-season management or genetic improvement, rather than solely increasing N to boost leaf area.

### 4.4 Nitrogen regulation of source-sink coordination

N fertilization can improve the source-sink relationship, significantly affecting the organ formation, morphological development, and yield composition of wheat (Duan et al., 2019; Tian et al., 2016). Significant differences in wheat yield and major agronomic traits have been observed under various N application conditions (Li et al., 2022; Tian et al., 2024). In this study, increasing N from 120 to 240 kg/hm^-2^ significantly enhanced leaf area, SLW, SPAD duration, and photosynthetic duration across all cultivars (**Figures 1**, **2**; **Tables 1**, **2**). While increased N fertilization significantly increases grain yield, kernels per spike, and spikes, of all cultivars (**Figures 3A, C, D**). However, the response was more pronounced in the source-limited cultivars (YM25 and ZM27). For these high-yielding genotypes, higher N supply mitigated source limitations by expanding the photosynthetic canopy and delaying senescence, thereby allowing the large sink potential to be more fully realized. This supports the strategy of matching N management with the specific source-sink type of the cultivar to optimize N use efficiency and yield. This study indicates that increasing N fertilizer application lowers the source-sink ratio (**Figure 6**). This suggests that the increase in leaf area due to N fertilization is greater than the increase in single-ear yield, thereby reducing source limitation. Therefore, increasing N fertilizer can alleviate source limitation and further enhance yield.

Correlation analysis reveals significant positive correlations between grain yield and spike yield, TKW, kernels per spike, spikes, grain volume, G_max_, leaf area, SLW, SPAD_max_, and Pn_max_ (**Figure 7**). This suggests that the increase in wheat grain yield is closely linked to the enhancement of source-sink performance. Additionally, leaf area and SLW are significantly positively correlated with yield and spike yield, TKW, kernels per spike, grain volume, G_max_, SPAD_max_, and Pn_max_ (**Figure 7**). This implies that increasing the source is crucial for improving grain sink capacity and sink activity.

Current research generally suggests that the yield potential of modern wheat cultivars still has room for improvement, but the limiting factor is shifting from the sink to the source, with the source becoming a constraint for further yield increases in the future (Gao et al., 2021; Zhang et al., 2016). Therefore, in order to meet the growing needs of sink capacity, improving source traits in future wheat breeding will be the key to further enhancing grain yield. The growth of leaf area in modern cultivars has now reached a bottleneck. To ensure adequate sink capacity, enhancing photosynthetic ability is the key to overcoming the current crop yield limitations and achieving high productivity. Increasing the source capacity can be achieved by improving the photosynthetic ability of wheat, including photosynthetic efficiency and canopy structure (Liang et al., 2010; Mason et al., 2013; Naruoka et al., 2012). Modifying the leaf canopy structure to allow the leaves to absorb more light energy is a key approach to improving photosynthetic capacity. Additionally, the source can be further enhanced by increasing chlorophyll content, photosynthetic rate, fluorescence parameters, Rubisco carboxylation capacity, soluble carbohydrate storage, and remobilization ability, thus maximizing the potential for wheat yield increase (Reynolds et al., 2012).

## 5 Conclusion

The superior grain yields of YM25 and ZM27 are attributed to their larger leaf area, higher SLW, extended SPAD, and photosynthetic duration (stay-green trait) post-anthesis. These traits ensure a robust source capacity that supports a high maximum grain-filling rate and superior grain fullness compared to the low-yielding cultivar YM1. Based on the coordination between source activity and sink strength, YM25 and ZM27 are classified as source-limited cultivars. Despite their strong source capacity, their exceptionally high sink potential (large kernels per spike and TKW) creates a high sink-source ratio, suggesting that further grain yield depends on enhancing photosynthetic efficiency. Conversely, YM1 is identified as a sink-limited cultivar, constrained by both low sink activity and insufficient source supply. N fertilization significantly optimizes source-sink dynamics by expanding the green canopy and delaying leaf senescence. This regulatory effect is particularly vital for source-limited cultivars, as it mitigates the assimilate deficit during the grain-filling stage, thereby maximizing the expression of their high-yield potential. In summary, achieving high wheat productivity requires the strategic selection of cultivars with high sink-source ratios and the implementation of precision N management to sustain source activity. These insights provide a theoretical framework for future breeding programs and optimized cultivation practices aimed at balancing carbon-nitrogen metabolism and source-sink interactions.

## Data Availability

The original contributions presented in the study are included in the article/**Supplementary Material**. Further inquiries can be directed to the corresponding author/s.
