# Supplementary material for: Source–sink characterization, classification and responses to nitrogen in different wheat cultivars
Source: Front Plant Sci. 2026 Apr 20;17:1817057. doi: 10.3389/fpls.2026.1817057 (PMC13136287; doi:10.3389/fpls.2026.1817057)
Supplement: Supplementary file 1 [file Table1.docx]

**Source–Sink Characterization, Classification and Responses to Nitrogen in Different Wheat Cultivars**

**Supplementary Table S1** Differences in leaf area and specific leaf weight (SLW) at anthesis in different wheat cultivars

| Cultivars | N rates | Flag leaf | | Top 2^nd^ leaf | | Top 3^rd^ leaf | |
| --- | --- | --- | --- | --- | --- | --- | --- |
|  |  | Leaf area(cm2) | SLW(g·m^-2^) | Leaf area(cm2) | SLW(g·m^-2^) | Leaf area(cm2) | SLW(g·m^-2^) |
| 2020-2021 | |  |  |  |  |  |  |
| YM1 | N240 | 24.75b | 37.40bc | 26.23bc | 39.04cd | 23.08bc | 36.45b |
|  | N120 | 20.63c | 35.87c | 22.67c | 36.51d | 20.96c | 32.52c |
| YM25 | N240 | 31.81a | 41.81a | 34.58a | 43.94a | 29.35a | 40.76a |
|  | N120 | 25.27b | 39.47ab | 28.78b | 40.93bc | 24.59b | 37.13b |
| ZM27 | N240 | 30.41a | 40.90a | 33.37a | 42.35ab | 28.36a | 39.45a |
|  | N120 | 24.65b | 39.35ab | 28.97b | 40.49bc | 23.45b | 35.37b |
| F-value | FN | 91.62** | 7.36* | 21.11** | 12.77** | 39.33** | 70.49** |
|  | FC | 39.92** | 14.28** | 21.76** | 16.79** | 23.06** | 31.96** |
|  | FN_×_C | 1.56 | 0.16 | 0.43 | 0.23 | 2.09 | 0.08 |
| 2021-2022 | |  |  |  |  |  |  |
| YM1 | N240 | 23.25b | 35.36bc | 25.38c | 37.54c | 20.33b | 33.14c |
|  | N120 | 19.01c | 33.73c | 23.17d | 35.45d | 17.88c | 30.80d |
| YM25 | N240 | 30.24a | 39.31a | 32.35a | 42.95a | 26.09a | 37.21a |
|  | N120 | 23.91b | 36.97ab | 26.63c | 39.24bc | 20.55b | 34.96b |
| ZM27 | N240 | 28.68a | 38.36a | 30.26b | 40.35b | 25.71a | 36.64a |
|  | N120 | 22.01b | 35.18bc | 25.75c | 38.05c | 19.42b | 33.45bc |
| F-value | FN | 88.80** | 14.74** | 68.12** | 30.78** | 163.31** | 36.46** |
|  | FC | 33.53** | 11.39** | 38.13** | 30.11** | 48.68** | 33.16** |
|  | FN_×_C | 1.56 | 0.52 | 4.20* | 1.09 | 9.91** | 0.48 |

Note: N240 and N120 respectively represent nitrogen application rates of 240 and 120 kg·hm^-2^. Values are means of three replicates. F-values were calculated by two-way ANOVA, * represents *p* < 0.05, ** represents *p* < 0.01, and different letters in a column indicate significant differences (*p* < 0.05), according to the LSD test. SLW: Specific leaf weigh

**Supplementary Table S2**  Differences in LAI in different wheat cultivars

| Cultivars | N rates | Jointing | Booting | Anthesis | Filling |
| --- | --- | --- | --- | --- | --- |
| 2020-2021 | |  |  |  |  |
| YM1 | N240 | 2.84a | 5.46b | 5.27b | 4.64b |
|  | N120 | 2.32b | 4.21d | 4.14d | 3.71c |
| YM25 | N240 | 3.28a | 6.42a | 6.24a | 5.46a |
|  | N120 | 2.23b | 4.83c | 4.74c | 4.30b |
| ZM27 | N240 | 3.03a | 6.16a | 6.22a | 5.22a |
|  | N120 | 2.25b | 4.88c | 4.67c | 4.40b |
| F-value | FN | 44.37** | 136.61** | 149.63** | 93.09** |
|  | FC | 0.78 | 17.99** | 19.97** | 19.99** |
|  | FN_×_C | 1.71 | 0.89 | 1.35 | 1.06 |
| 2021-2022 | |  |  |  |  |
| YM1 | N240 | 2.66a | 5.23b | 5.19b | 4.22b |
|  | N120 | 2.23b | 4.11d | 4.06d | 3.22d |
| YM25 | N240 | 2.92a | 6.00a | 5.84a | 5.16a |
|  | N120 | 2.22b | 4.71c | 4.53c | 3.683c |
| ZM27 | N240 | 2.70a | 5.92a | 5.80a | 4.95a |
|  | N120 | 2.26b | 4.67c | 4.56c | 3.71c |
| F-value | FN | 25.58** | 119.31** | 166.53** | 186.14** |
|  | FC | 0.52 | 15.47** | 15.29** | 23.46** |
|  | FN_×_C | 0.73 | 0.21 | 0.30 | 2.26 |

Note: N240 and N120 respectively represent nitrogen application rates of 240 and 120 kg·hm^-2^. Values are means of three replicates. F-values were calculated by two-way ANOVA, * represents *p* < 0.05, ** represents *p* < 0.01, and different letters in a column indicate significant differences (*p* < 0.05), according to the LSD test.

**Supplementary Table S3** The differences of spike characters in different wheat cultivars

| Cultivars | N rates | Spike lengh (cm) | Spikelet number | Kernels per spikelet | Spikelet density  (cm^-1^) | Spike yield  (g·spike^-1^) |
| --- | --- | --- | --- | --- | --- | --- |
| 2020-2021 | |  |  |  |  |  |
| YM1 | N240 | 10.33b | 19.98ab | 2.16d | 3.61e | 1.44d |
|  | N120 | 8.50c | 18.78bc | 2.26c | 4.08c | 1.29d |
| YM25 | N240 | 11.63a | 20.61a | 2.50a | 4.01c | 2.15a |
|  | N120 | 9.09c | 18.27c | 2.55a | 4.40a | 1.82bc |
| ZM27 | N240 | 11.85a | 20.53a | 2.39b | 3.73d | 2.00ab |
|  | N120 | 9.07c | 18.26c | 2.51a | 4.28b | 1.68c |
| F-value | FN | 93.79** | 34.328** | 14.58** | 273.91** | 17.72** |
|  | FC | 7.35** | 0.01 | 67.14** | 55.08** | 34.50** |
|  | FN_×_C | 1.35 | 1.25 | 0.88 | 3.03 | 0.98 |
| 2021-2022 | |  |  |  |  |  |
| YM1 | N240 | 8.87b | 19.60a | 2.19e | 4.06d | 1.38de |
|  | N120 | 7.43c | 17.60b | 2.28d | 4.43c | 1.22e |
| YM25 | N240 | 10.05a | 19.95a | 2.48b | 4.43c | 1.98a |
|  | N120 | 8.04c | 17.93b | 2.55a | 4.84a | 1.68bc |
| ZM27 | N240 | 10.44a | 20.02a | 2.40c | 4.10d | 1.84ab |
|  | N120 | 8.06c | 17.81b | 2.42c | 4.59b | 1.51cd |
| F-value | FN | 113.29** | 77.83** | 28.27** | 412.22** | 23.92** |
|  | FC | 13.57** | 0.859 | 199.41** | 125.19** | 34.27** |
|  | FN_×_C | 2.22 | 0.09 | 2.81 | 2.93 | 1.03 |

Note: N240 and N120 respectively represent nitrogen application rates of 240 and 120 kg·hm^-2^. Values are means of three replicates. F-values were calculated by two-way ANOVA, * represents *p* < 0.05, ** represents *p* < 0.01, and different letters in a column indicate significant differences (*p* < 0.05), according to the LSD test.

**Supplementary Table S4** The differences of grain characters in different wheat cultivars

| Cultivars | N rates | Grain length (cm) | Grain width (cm) | Grain thickness  (cm) | Grain volume  (μl) | Fullness  (g·ml^-1^) |
| --- | --- | --- | --- | --- | --- | --- |
| 2020-2021 | |  |  |  |  |  |
| YM1 | N240 | 0.62d | 0.31c | 0.25c | 49.48b | 0.79c |
|  | N120 | 0.57e | 0.27d | 0.22d | 48.46b | 0.77c |
| YM25 | N240 | 0.74a | 0.45a | 0.35a | 56.14a | 0.82a |
|  | N120 | 0.71bc | 0.41b | 0.32b | 55.86a | 0.81ab |
| ZM27 | N240 | 0.73ab | 0.44ab | 0.34ab | 56.23a | 0.81b |
|  | N120 | 0.68c | 0.42b | 0.32b | 55.10a | 0.80b |
| F-value | FN | 26.74** | 20.93** | 23.00** | 0.84 | 5.52* |
|  | FC | 114.35** | 158.44** | 154.22** | 26.74** | 64.34** |
|  | FN_×_C | 0.35 | 0.91 | 0.30 | 0.09 | 0.44 |
| 2021-2022 | |  |  |  |  |  |
| YM1 | N240 | 0.64d | 0.33d | 0.26d | 48.31cd | 0.80d |
|  | N120 | 0.57e | 0.29e | 0.23e | 46.19d | 0.80c |
| YM25 | N240 | 0.82a | 0.45a | 0.37a | 53.56a | 0.83a |
|  | N120 | 0.78b | 0.41bc | 0.34bc | 52.67ab | 0.82b |
| ZM27 | N240 | 0.77bc | 0.43b | 0.36ab | 52.61ab | 0.82b |
|  | N120 | 0.74c | 0.39c | 0.33c | 49.84bc | 0.82b |
| F-value | FN | 27.59** | 26.95** | 24.14** | 6.72* | 0.027 |
|  | FC | 183.57** | 120.82** | 150.75** | 21.57** | 54.83** |
|  | FN_×_C | 2.40 | 0.03 | 0.25 | 0.54 | 11.10** |

Note: N240 and N120 respectively represent nitrogen application rates of 240 and 120 kg·hm^-2^. Values are means of three replicates. F-values were calculated by two-way ANOVA, * represents *p* < 0.05, ** represents *p* < 0.01, and different letters in a column indicate significant differences (*p* < 0.05), according to the LSD test.
